# Supplementary figures and images for: Transparent number-naming system gives only limited advantage for preschooler's numerical development: Comparisons of Vietnamese and French-speaking children
Source: PLoS One. 2020 Dec 7;15(12):e0243472. doi: 10.1371/journal.pone.0243472 (PMC7721146; doi:10.1371/journal.pone.0243472)

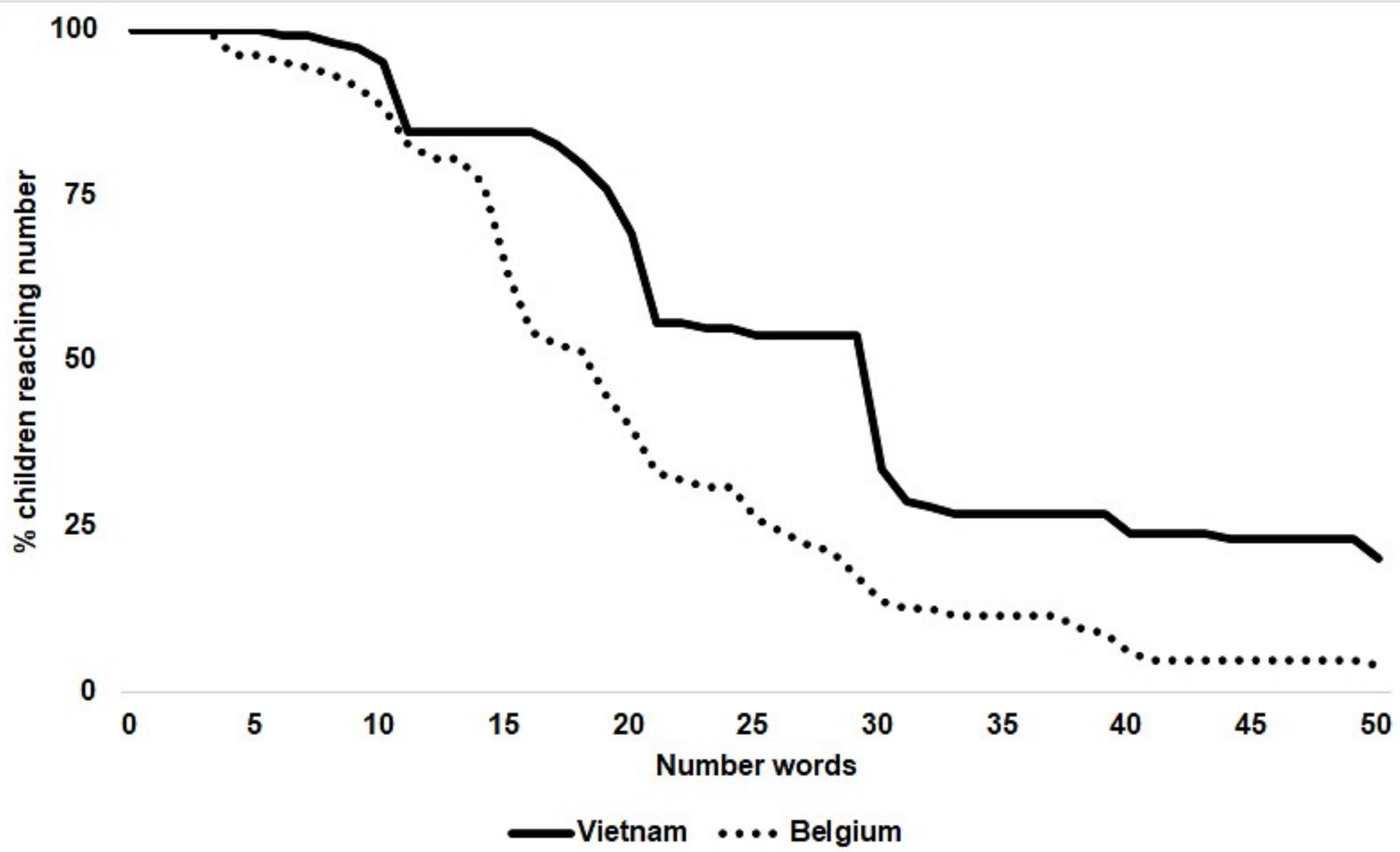

Supplement: S1 Raw image — (PDF) [file pone.0243472.s001.pdf]
