# Supplementary material for: Transparent number-naming system gives only limited advantage for preschooler's numerical development: Comparisons of Vietnamese and French-speaking children
Source: PLoS One. 2020 Dec 7;15(12):e0243472. doi: 10.1371/journal.pone.0243472 (PMC7721146; doi:10.1371/journal.pone.0243472)
Supplement: Attachment — Submitted filename: Response to Reviewers.docx [file pone.0243472.s012.docx]

**The Impact of Language on Numerical Development: Dissociation between Counting and Cardinality in Vietnamese and French-Speaking Preschoolers**

*** Author's reply in blue**

**Editor**

Thank you for submitting your manuscript to PLOS ONE. I have sent your manuscript to two expert reviewers and have now received their comments back. As you can see attached and below, both reviewers are positive about your study and highlight the uniqueness and interesting aspect of your sample. I agree with them. Nonetheless, both reviewers also point to several important issues that I think you would need to address in a major revision. I will not reiterate all of the reviewers' points here, as the reviewers made them very clear. But I concur with the reviewers that you run a large number of analyses in the manuscript, which raises a multiple comparison issue that might be addressed with adequate corrections or a change in analysis strategy (see suggestions from reviewer #2). Both reviewers also highlight that Bayesian analyses might help you make the most of null results and I also think that this might be very informative. Therefore, I encourage you to address these issues (as well as all of the other concerns from the reviewers) and submit a revised version of your manuscript. I will send this version back to the original reviewers.

Dear Editor,

We thank the Editor for your favorable view and your positive assessment of our work.

We corrected for the more robust analysis (MANCOVA, Bayesian analysis, Moderation analysis, and Power analysis) and reduced many unnecessary analyses. We integrated the corrections required by the Editor, Reviewer #2, and Reviewer #1 in the revised manuscript (with tracked changes). All these modifications are detailed in the following point-by-point reply to the reviewers' comments.

Because there are some changes in the research results (i.e., effects of nationality on cardinal knowledge disappear after correction by Mancova), we suggested correcting the abstract and the title of the article.

The new title suggested is: " **Transparent number-naming system gives only limited advantage for preschooler's numerical development: Comparisons of Vietnamese and French-speaking children.**"

**Reviewer #2**

In this manuscript, the authors present cross-cultural data that reveals that Vietnamese children outperformed Belgian children in counting, but not in other symbolic and nonsymbolic tasks, with Belgian children actually outperforming Vietnamese children in cardinality knowledge. Additionally, this research reports that Vietnamese children tend to be more stimulated in the home environment. First, I wish to note that this is a very impressive dataset and I applaud the authors for collecting such a large cross-cultural sample. I think that this is an interesting and important research question and has the potential to add to the current state of our understanding of the influence of language on number processing in young children. However, I have many concerns regarding the methodological and statistical choices as well as several concerns about the theoretical setup and interpretation of this data. Below I outline both major and minor concerns.

Dear Reviewer #2

We thank the Reviewer for your positive comments and several valuable suggestions. Please see our following answers to your comments.

Major Issues
Methodological
1. This manuscript contains many different analyses (my estimate: 12 ANOVAS, 10 Chi-squared tests, 13 t-tests, 24 correlations, 2 regressions). However, I don't see any correction for multiple comparisons. I recommend reducing the number of analyses included where possible and including fewer, but more sophisticated analyses. I recommend analysing the cognitive and numerical data using a MANOVA, GLM or perhaps a mixed effects model, instead of multiple univariate ANOVAs. Similarly, rather than running correlation analyses on the full sample and each country separately to examine the relation between numerical ability and home numeracy, I recommend running moderation analyses. In the cases where multiple analyses are necessary, corrections for multiple comparisons should be included.

Yes. We corrected the multiple comparisons by MANCOVA and Moderation analyses for the relation between numerical ability and home numeracy. On the other hand, we removed some unnecessary Chi-squared, *t*-test related size effect analyses on numerical tasks (enumeration, collection comparison, number words comparison, and approximate addition) and *t*-test for each item in home numeracy questionnaire. Please see the updated results section (page 22-32).

2. I have concerns about the decision to use median splits for continuous data, particularly with age (but also education level). There are statistical issues with converting continuous data into categories in general (see: <https://www.theanalysisfactor.com/continuous-and-categorical-variables-the-trouble-with-median-splits/>). It is particularly problematic for age given the dramatic changes observed in basic number processing during the age range included in the sample (e.g. Le Corre & Carey, 2007; Merkley & Ansari, 2016). The authors should use one of the analyses methods suggested above, with age included as a continuous predictor.

We thank the Reviewer for this insight. We agree with your suggestion. Therefore, age (in months) and education level (i.e., number of schooling years) were considered as continuous variables and age was introduced as a covariate in most analyses.

3. Many of the key conclusions drawn from this data are a result of null findings (i.e. that Belgian and Vietnamese children do not differ). Critically, the frequentist-based statistics used in the current study can only be used to reject the null hypothesis. They cannot be used to confirm that there is in fact no difference between groups. Therefore, in order to draw key conclusions (e.g. "the performance of the children in both countries with equivalent" – line 659), the authors should conduct and include follow-up Bayesian analyses to test if the null hypothesis is true. In line with this, in order to conclude that nationality does indeed improve the hierarchical model, a model comparison method (again Bayesian would do the trick here), is needed to compare the models.
Yes. We thank the Reviewer for the suggestion. We used JASP for Bayesian analyses to test if the null hypothesis is correct (lines 548-549). We have added these analyses for the results section (page 25-26).

Theoretical
4. The authors indicate that Vietnamese parent's attitudes towards academic success are more similar to the European view, making French-Belgium and Vietnamese's children ideal groups to compare. However, these statements are not supported by empirical evidence. I think the introduction would benefit from a stronger theoretical setup with supporting citations explaining why these two groups are ideal to compare.

We thank the Reviewer for bringing this point to our attention. The lack of assessing the parental attitude toward academic success was acknowledged as a limitation of our current study (page 4, lines 922-923)

We propose four reasons to support that these two samples were quite comparables:

(1) The samples were preschoolers, an age that is less affected by formal schooling

(2) There was an identical distribution of gender ratio in the two samples

(3) All children are monolingual speakers; Belgian children in Wallonia region only speak French, while Vietnamese children only speak Vietnamese

(4) The similarity in parents' education level (based on the number of years of schooling as a continuous variable) was quite similar

Although we did not measure parent's attitudes towards academic success, we measured parents' stimulation of children's learning through activities related to reading, counting, reading numbers, etc., i.e., activities related to numeracy or literacy. Our results show that although Vietnamese parents stimulated their child more on home numeracy and home literacy activities than Belgian parents, this effect is moderate. Moreover, the average frequency reported in the Vietnamese group is around 2, which means "sometimes". By contrast, Chinese parents are often reported to spend time each day monitoring the academic activities of their children (Chua, 2011).

In an internal study conducted on parents with children under ten years old in Vietnam (N = 308) in two major cities, Hanoi, and Ho Chi Minh City, and in others provinces showed that most parents (74%) expect their children to gain hight education level in the future, typically "*above bachelor*." When comparing among cities and provinces, it is clear that parents from Hanoi (80.7%), had higher expectations than parents from Ho Chi Minh city and from other provinces (WS-Group-Market-Research, 2012). The level of parental expectations varies by racial/ethnic group (Yamamoto & Holloway, 2010). Although currently, there is a lack of studies on Vietnamese parents' expectations level for children's academic success, the most survey found that Chinese parents put the most pressure on students. For instance, in an international comparison study with Japan, India, and the U.S., they reported too many expectations in 63% of the sample for China versus Japan (59%), India (51%), U.S. (15%)) [1]. Roughly more than six-in-ten Chinese (63%) say all this pressure is too much, while 20% feel the demands are about right. Only 11% say students need more parental pressure. Mainstream discussions in parenting style and parental pressure on children is about Chinese tiger moms, who were considered as a profile a perceived psychological toll resulting from high parent expectations on children (Chua, 2011). However, this problem was rarely reported in Vietnam.

Although parental expectations for children's education in Asia are usually higher than in Europe (Hunt, 2005), a change has been observed since the economic reforms in 1986 (i.e., Doi Moi policy) when Vietnam embarked on economic liberalization and a transition to market socialism as it also led to more access to western cultures and beliefs (Kim et al., 2009). These changes also brought new trends of parenting (with less use of corporal punishment and more acceptance of some individualistic values appear more commonly; Mestechkina, Son & Shin, 2014). Besides, some cultural similarities in southern Vietnam and French culture are influenced by the French colonial regime. Despite the contrasts of collectivism between Asia and individualism in Europe, Vietnam was a French colony for 61 years (1884-1945), so it was also influenced by the French culture. In particular, our sample group is based in Ho Chi Minh City/ (ancient name: Saigon) in southern Vietnam. Previous research (Różycka-Tran et al., 2017) on 511 Saigon people, 538 Hanoi people, showed that "*People in Saigon attributed significantly more importance to the hedonism, stimulation and power-achievement values. Only in Hanoi (capital in Vietnam), benevolence was the most preferred value, the same as the security-conformity-tradition value* ". Based on Schwarts's value system theory, hedonism and stimulation are fundamental values in a European country (Bilsky et al., 2011).

*Reference:*

Bilsky, W., Janik, M., & Schwartz, S. H. (2011). The structural organization of human values – Evidence from three rounds of the European Social Survey (ESS). Journal of Cross-Cultural Psychology, 42, 759-776. <http://dx.doi.org/10.1177/0022022110362757>.

Hunt, P. C. (2005). An introduction to Vietnamese culture for rehabilitation service providers in the United States. In J. H. Stone (Ed.), Culture and disability: Providing culturally competent services (pp. 203–223). Thousand Oaks: Sage.

Mestechkina T., Son N.D., Shin J.Y. (2014) Parenting in Vietnam. In: Selin H. (eds) Parenting Across Cultures. Science Across Cultures: The History of Non-Western Science, vol 7. Springer, Dordrecht. <https://doi.org/10.1007/978-94-007-7503-9_5>

Kim, B. S. K., Ahn, A. J., & Lam, N. A. (2009). Theories and research on acculturation and enculturation experiences among Asian American families. In N. T. Trinh, Y. C. Rho, F. G. Lu, & K. M. Sanders (Eds.), Handbook of mental health and acculturation in Asian American families (pp. 25–43). New York: Humana Press.

Różycka-Tran, J., Ha, T. T. K., Cieciuch, J., & Schwartz, S. H. (2017). Universals and specifics of the structure and hierarchy of basic human values in vietnam. Health Psychology Report, 3(3), 193-204. doi:10.5114/hpr.2017.65857

Yamamoto, Y., & Holloway, S. D. (2010). Parental expectations and children's academic performance in sociocultural context. Educational Psychology Review, 22(3), 189-214. doi:10.1007/s10648-010-9121-z

<https://www.slideshare.net/WS-Group-Market-Research/survey-on-parents-orientation-and-expectation-for-their-children-in-vietnam-2012>

[https://www.pewresearch.org/global/2006/08/24/parental-pressure-on-students/]

5. Relatedly, I have several questions about the comparability of the samples.
a. Specifically, the authors indicate that the two groups have equivalent SES. This is based on years of education. My question is whether equivalent years of education results in similar occupational levels in both cultures? Additionally, as the samples are selected to be equivalent from larger samples, I am curious about where the Belgian and Vietnamese samples each fall within the context of the larger sample. More specifically, while the years of education between samples is reported as approximately equivalent, it is possible that one sample has low SES compared to the larger within-country sample and the other has high SES compared to the larger within country sample. A difference in the relative position within the broader community should be checked for equivalence and potentially discussed.
b. Is it typical for individuals in each country to be monolingual? For example, is it common for children in Belgium to only speak French or does this community typically speak French and Belgian? Is there a difference in the degree to which children in each country are typically exposed to other languages?
c. I think that the authors made a great decision to test children at the preschool age, prior to formal schooling, to reduce the potential influence of differences in the educational system! The authors note that children who had additional math tutoring were excluded, but I did not see a report of the number of children enrolled in some sort of preschool or daycare. I think the number of children enrolled in preschool/daycare should be reported. If many children are enrolled in preschool, it could be important to consider whether each country has a preschool curriculum that is followed and how they differ.

a. Unfortunately, we lack SES information; this is now added in the discussion section (page 40, line 918). In a densely populated country like Vietnam (over 90 million population) and a small country like Belgium (with over 11 million population), we have chosen the locations where the parental education level could be the most similar. Vietnam's rapid economic growth in the last 30 years has transformed it from one of the world's poorest countries to a middle-income country (World Bank, 2013). In particular, the global SES has changed importantly in cities. Accordingly, we selected mainly children in Ho Chi Minh City and Binh Duong as two developed cities in southern Vietnam, of which Ho Chi Minh is Vietnam's economic focal point. In Belgium, we choose French-speaking cities, all of which are big cities like Ottignies, Namur, Charleroi, but where the SES level is average for the country. We have added this information in the introduction section (page 9, lines 222-231)

Parental educational level is an important predictor of children's educational and behavioral outcomes (Davis-Kean, 2005). Davis-Kean (2005) found direct effects of parental education, but not income, on European American children's standardized achievement scores; both parental education and income exerted indirect effects on parents' achievement-fostering behaviors, and subsequently children's achievement, through their effects on parents' educational expectations.

As explained in the updated participants section (page 13-14), both mothers and fathers in each sample have an average of 15 years of schooling, which is equivalent to college education level (in Vietnam) or superior education (in Belgium). Most parents had studied from College or Superior education to university and above (79 VN and 85 BEL mothers, 82 VN and 74 BEL fathers). Years of education results in similar occupational levels in both cultures. For example, in order to find a good job, the condition is to have a university degree or higher, both in Vietnam and Belgium.

Reference:

World Bank.2013.*World development indicators 2013 (English).* Washington, D.C. : World Bank Group. http://documents.worldbank.org/curated/en/449421468331173478/World-development-indicators-2013

Davis-Kean, P.E. (2005). The influence of parent education and family income on child achievement: The indirect role of parental expectations and the home environment. *Journal of Family Psychology*;19:294–304.

b. In Belgium, there are three regions (French-speaking Wallonia, Dutch-speaking Flanders, and the Brussels capital speaking both French and Dutch). In our sample, Belgian children came from the Francophone Wallonia region and speak only French (although they will learn an another language later on in school, usually around 10 y.o.). Children in Vietnam are Vietnamese and speak only Vietnamese.

c. We thank the Reviewer for the comment. Children who take extra mathematics courses outside of school were excluded from the study (*n* = 23 VN). We added this information in the participant section (page 13, ligne 318 -319). We think it is a small amount (6.86%) among the initial sample (*N* = 335) in which 310 children used in a longitudinal study of Vietnamese children, 23 children who had additional math tutoring, and 2 children whose parent do not report about additional math's participation. We emphasize that children taking additional math courses (e.g., Kumon, Mathnasium) are training programs at education centers outside the preschool (i.e., independent of preschool). Children only attend weekends or after school hours to preschool. Therefore, the preschool curriculum in Vietnam and Belgium did not differ. Notably, the educational program in Vietnam is identical for all areas and all private and public schools because they follow the same rule of Vietnam's Education and Training Ministry that there is no teaching of letters and numbers before grade one of primary school. In Belgium, similarly, students do not study letters and numbers before entering grade 1. On the other hand, in Belgium, there is no extra math program outside the preschool, like in Vietnam.

**Minor Issues**
Theoretical
6. I would appreciate a more comprehensive overview of the process of acquiring symbolic number knowledge (e.g. the process of learning how to count; acquiring cardinal knowledge; distinction between procedural and conceptual knowledge).

Yes. A new section consisting in a quick overview of numerical development has been added in the introduction section (page 2-3)

7. The authors report one study (ref 31) where symbolic and nonsymbolic number processing influence each other. However, as this is a widely studied and debated topic in the field of numerical cognition. Since symbolic and nonsymbolic numerical knowledge are included and compared as variables of interest, I think the introduction could benefit from a slightly more comprehensive description of this area. I think this could also be helpful for setting up clearer predictions for symbolic and nonsymbolic number processing.

Indeed, there are large debates on these issues, and we wanted to respect the size limitation for the paper. Following this remark, we have added some other references regarding the impact of learning symbolic math on the ANS; please see in the introduction section (page 5, lignes 118-137)

References:

Piazza, M., Pica, P., Izard, V., Spelke, E. S., & Dehaene, S. (2013). Education enhances the acuity of the nonverbal approximate number system. Psychological Science, 24, 1037–1043. <https://doi.org/10.1177/0956797612464057>

Pica, P., Lemer, C., Izard, W., & Dehaene, S. (2004). Exact and approximate arithmetic in an Amazonian indigene group. Science, 306, 499–503

Dowker, A., & Roberts, M. (2015). Does the transparency of the counting system affect children's numerical abilities? Frontiers in Psychology, 6. https://doi.org/10.3389/fpsyg.2015.00945

8. In the discussion, the authors note that there are differences in the kinds of home numeracy done in each country (formal vs informal). I thought this was an interesting and wonder if it should be included as an exploratory section in the results? I don't feel strongly about this and leave it up to the authors!

Yes. Thank the Reviewer for this suggestion. We analyzed differences in the kinds of home numeracy done in each country (formal vs. informal) with two *t*-tests for these two types and reported that in an exploratory result section (page 29-30, lignes 686-709).

To sum, Vietnamese parents stimulated their child more on formal numeracy activities than Belgian parents. However, the frequency of informal numeracy activities did not differ between those of Vietnamese parents and Belgian parents. The Bayesian analysis also supported the significant difference in formal numeracy and the absence of difference in nonformal numeracy.

Methodological
9. While the sample size seems sufficient to detect at least a medium effect size within the analyses included (and is impressive!), it would be helpful to include a power analysis to indicate what exact effect sizes can be detected with each analysis using the current sample.

Yes. According to the suggestion of the Reviewer, we conducted post hoc power analyses using GPower main analysis (two MANCOVAs for numerical tasks and repeated-measure ANCOVA for home numeracy) to detect medium effects (*f^2^=* 0.25) with 95% power using a MANCOVA with alpha at .05 and medium effects (*f^2^=* 0.0625) with 95% power using a repeat-measure ANCOVA (page 23, lignes 563-568). Results of power analysis were added in three locations (page 25, lignes 596-598 and lignes 604-607); page 29, lignes 682-685)

10. The decision to include size as a within-subjects variable, grouped into two levels, needs further justification.

As you decided to run MANOVAs instead of multiple ANOVAs, the size effect was not included anymore in the analyses. This did not change much of the results as the previous analyses, including that factor, mostly failed to show any significant interaction between Nationality and Size. (Please see page 23, lines 554-557)

11. The Huynh-Feldt correction for sphericity is the least conservative correction and often considered to be too liberal and consequently overestimates sphericity. I am curious why the authors did not choose the more typical Greenhouse-Geisser? Unless epsilon is >.75, I recommend the authors switch to Greenhouse-Geisser (Girden, 1992).

We thank the Reviewer for the insight to apply in further study. The new analysis was run on univariate repeated-measures designs as before but mainly with 2 MANOVAs. Therefore, the old interpretations related to The Huynh-Feldt correction for sphericity were removed.

12. A more complete description of the give me a number task would be helpful. For example, did the child need to correctly give-N twice at one number and incorrectly give-N twice at the number above to be considered that knower-level? Generally, did the authors use the traditional protocol (from Wynn, 1992)? If so, it would be best to cite the original paper. Also, I think the give me a number task is most typically referred to as the "Give-A-Number Task" or "Give-N", rather than Give me a number. This isn't super important but using the most common name might make it easier to find this article for those looking at this task.

We appreciate the suggestion to refer to the Give-N task. We corrected it in the manuscript.

In the Methodology section (page 17, line 411-414), we add the complete description of the stopping rule of the Give-N.

Yes, if the child correctly gives N twice for a certain number and is incorrect in giving N+1 twice, then N is considered the child's knower-level

We apply this rule when children are unsuccessful at any number (whether small or large). Please see the example below: when the child was unsuccessful with Give 4, the child must return to the previous item (Give 3). After a successful answer at Give 3, the child will return the next item (Give 4). If the child again fails in Give-4, the task is stopped. The child's knower-level is three.

| **Item** | **Notation** | | |
| --- | --- | --- | --- |
|  | **Testing** | **Return 1** | **Return 2** |
| Give 1 | 0 - **1** | 0 - 1 | 0 - 1 |
| Give 2 | 0 - **1** | 0 - 1 | 0 - 1 |
| Give 3 | 0 - **1** | 0 - **1** | 0 - 1 |
| Give 4 | **0** - 1 | **0** - 1 | 0 - 1 |
| Give 5 | 0 - 1 | 0 - 1 | 0 - 1 |
| Give 6 | 0 - 1 | 0 - 1 | 0 - 1 |
| **Max objects with at least two successful answer** | | | **3** |

In the description, we explained: This task was modeled on the one used by Negen and Sarnecka (2015) to assess children's understanding of cardinality knowledge (i.e., the cardinal meaning of number words, see Wynn, 1992). The protocol is from Negen and Sarnecka (2015). The difference between the two protocols is: in Wynn's protocols, the child was invited to give a puppet one to five items. However, in Negen and Sarnecka's protocol, the child was invited to give a rubber toy one to six items. Besides, in Wynn's (1990, 1992) version, children need to answer the question Is that N? (i.g., for a trial (give 2 puppets) after children gave 2 puppets, the experimenter asks is that 2 pubbets?). In the Negen and Sarnecka's version, there are no recount and no follow-up questions (see Krajcsi et al., 2018). Krajcsi et al. (2018) showed that the recount version displays higher performance. Thus, we chose the version without recount and follow-up questions.

Krajcsi, A., Fintor, E., & Hodossy, L. (2018). A refined description of preschoolers' initial symbolic number learning. https://doi.org/10.31219/osf.io/2kh9s

13. As mentioned above, I do not follow the logic to conduct a median split on the education data and use a chi-squared test to compare years of education. Why did the authors choose this method rather than to keep years of education as a continuous variable and then run a t-test to compare years of education?

Along with the Reviewer's comments, we now have considered the age and years of education as a continuous variable instead of group variables.

Stylistic
14. The figures are nice, but it would really great to see the data points plotted under the line (or violin plots) to understand not only the mean but also variability within the two samples. I leave this up to the authors.

We appreciate the suggestion. However, after corrected for the new analysis with MANOVAs and eliminated many Chi-square, we suggested Table 2, which shows very clearly the performance's average. Therefore, the figure showing the average across countries was deleted (as recommended by Reviewer #1).

15. The reporting of nonsignificant findings is inconsistent. Sometimes it is reported Is the f<1 and other times the nonsignificant pvalue is reported. I think the authors should stick to one of the two reporting styles.

Yes. We corrected for *p*-value in the manuscript and report fully for *F* instate of *F* < 1.

16. The whole manuscript would benefit from a thorough proofread for grammar and clarity. In particular, there is a lot of phrasing that seems a bit odd and switching between tenses. A few examples of phrases that would benefit from editing include:
o "in the same way" – Line 137
o "Seems to be quite a significant difference" Line 160
o "The prediction… is still an open question" Lines 220-221
o "According to Chen and Li (2014), there would be a…" – Line 222
o "Belgian children performed not significantly better" – Line 543

Yes. Thank the Reviewer for your attention. We rephrased these sentences (lines 284), and some previous sentences were deleted (ligne 137, 160, 222, 543). The whole manuscript was edited by Grammarly solfware and then edited by a person from English proofreading from UCLouvain.
o "There was a small interaction…" – Line 656

Yes. We deleted this sentence because of the exclusion of size-related analysis.

**Reviewer 1**

The study aims at investigating whether the way number words are formed influences early numerical processing. While Vietnamese number words are highly transparent, French number words are not (e.g., teen, tens). The authors tested a carefully matched sample of French-speaking Belgian and Vietnamese children aged 3.5 to 6 (thus with minimal previous exposure to formal schooling) on several numerical tasks. To differentiate more general cultural differences between the language groups from actual language-related influences, the authors chose Vietnam (as former French colony) instead of another less culturally comparable Asian sample and additionally assessed the home learning environment via a parent questionnaire. Results seem to be compatible with the idea that language influences certain aspects of early numerical knowledge acquisition. Language might play a role when it comes to the acquisition of the number word sequence but less so regarding a more profound conceptual understanding of number words (e.g. cardinality).

I very much enjoyed reading the manuscript. It is clearly written, relevant literature is presented, the current study is nicely embedded in previous research. I especially like the carefully chosen sample and the comprehensive set of measures. I have several comments/questions (see below) which I believe can be addressed in a minor revision of the current manuscript.

Dear Reviewer #1,

We appreciate the positive assessment of our work, and thank you so much for several useful suggestions.

\# Introduction

The authors nicely outline results of relevant studies investigating cross-cultural differences in early years (~p.3). In my view it would be important to add information on whether or not the presented results actually point to between-culture/country differences or indeed to specific language/number-word differences.

Without language-specific effects (e.g. something like "children with less transparent teen-numbers commit more errors for teen numbers but not for other (transparent) numbers compared to children with fully transparent numbers") it is hard to fully disentangle culture and language in many cases. The second paragraph deals with this issue to a degree, it might be nice to refer back to the first section and give an example on how results might be interpreted differently (both language and teaching practices/home environment might lead to the same between country differences).

Yes, we added the introduction about the difficulty (i.e., to differentiate more general cultural differences between the language groups from actual language-related influences) in this area's research (page 8, lines 191-205), and then explained how our research tried to overcome this problem in the present study section (page 8-9, lines 210-218).

It would also be helpful if the authors explained more explicitly how the tried to overcome this problem in the present study (Vietnam is culturally closer than other Asian countries -> minimize differences, evaluate home environment -> identify potential confound, look at effects on item level (e.g. for counting one wouldn't expect differences for numbers up to 10 but if language matters for larger numbers we should see differences when number word structures differ).

Yes. We thank the Reviewer for this suggestion. We explained more precisely how we tried to overcome this problem in the current study (page 8-9) and re-formulated the hypothesis of the study (page 11, lines 277-279)

#Methods

Sample: This seems to be a very nice and carefully chosen sample!

p. 10: "The highest level of parents' education was reported. This information was then encoded into the number of years of study because this factor was a little different between Vietnam and Belgium." I do not fully understand what the authors mean by this.

We thank the Reviewer for the comment. We corrected that in the participant's section (page 13-14, lines 327-352)

The highest level of parents' education was reported. This information was then encoded into the number of years of schooling because this factor was a little different between Vietnam and Belgium. For instance, primary education is five years in Vietnam but six years in Belgium; then, the secondary education level in Vietnam includes seven years, while in Belgium, it is six years. University in Vietnam is for four years and independent with Master (2 years), while in Belgium, University degrees are usually five years, a Bachelor (3 years), and a Master (2 years) levels. So, for instance, the number of years of schooling is 12 in both Belgium (6+6) and Vietnam (5+7) for a parent who has completed both primary and secondary education. (see Table 1)

| **Table 1. The education system in Vietnam, Belgium, and the number of schooling years according to education level.** | | | | | | | |
| --- | --- | --- | --- | --- | --- | --- | --- |
|  | **Vietnamese Education System** | | |  | **Belgium Education System** | | |
| Level | Age | *N* years | *N* Years of Schooling | Level | Age | *N* years | *N* Years of Schooling |
| **1. Primary education** | 6 to 11-y.o | 5 | 5 | **I. Primary education** | 6 to 12-y.o | 6 | 6 |
| **2. Lower Secondary education** | 12 to 16-y.o | 4 | 9 | **II. Secondary education** | 12 to 18-y.o | 6 | 12 |
| **3. Upper Secondary education** | 16 to 18-y.o | 3 | 12 |  |  |  |  |
| **4. Professional training** | 18 to 20-y.o after level 3 | 2 | 14 | **III. Superior education** | 19 to 21-y.o | 3 | 15 |
| **5. College** | 18 to 21-y.o after level 3 | 3 | 15 |  |  |  |  |
| **6. University** | 18 to 22-y.o after level 3 | 4 | 16 | **IV. University  (Bachelor & Master)** | after level III | 5 | 17 |
| **7. Master** | after level 6 | 2 | 18 |  |  |  |  |
| **8. Doctor of Philosophy** | after level 7 | 4 | 22 | **V. Doctor of Philosophy** | after level IV | 4 | 21 |

Give-N task: "For example, children who succeeded at one and two, but failed at three, were called *two-knowers*. Children who had at least twice as many successes as failures for trials of five and six were called cardinal principle knowers (i.e., C.P. knowers). The highest number of objects the child reached correctly was used as the child's knower level. "

I am curious about the stopping rules the authors applied for this task. From my own experience with this task I can say that it is sometimes tricky to meet the "twice as many successes as failures" criterion when testing a child (because this might results in a really high number of trials and children then might lose interest for example). Did you apply this rule only for 5 and 6? And for smaller numbers it was enough to give it correctly once? (Not sure if I fully get the description)

Yes. We thank the Reviewer for bringing this point to our attention.

In the Methodology section (page 17), we add the complete description of the stopping rule of the Give-N task as follows: the task stopped when a child made two mistakes on the same number N or when they arrived at the last item (number 6). We apply this rule when children are unsuccessful at any number (whether small or large).

Please see the example below: when the child was unsuccessful with Give 4, the child must return to the previous item (Give 3). After a successful answer at Give 3, the child will return the next item (Give 4). The Give-4 fails a second time; therefore, the task was stopped. The child's knower-level is three-knower. When we practice with children, we create an atmosphere like a game, so they do not get bored.

| **Item** | **Notation** | | |
| --- | --- | --- | --- |
|  | **Testing** | **Return 1** | **Return 2** |
| Give 1 | 0 - **1** | 0 - 1 | 0 - 1 |
| Give 2 | 0 - **1** | 0 - 1 | 0 - 1 |
| Give 3 | 0 - **1** | 0 - **1** | 0 - 1 |
| Give 4 | **0** - 1 | **0** - 1 | 0 - 1 |
| Give 5 | 0 - 1 | 0 - 1 | 0 - 1 |
| Give 6 | 0 - 1 | 0 - 1 | 0 - 1 |
| **Max objects with at least two successful answer** | | | **3** |

Overall regarding tasks with two-digit numbers: I was wondering if the authors always presented all items to the children. I could imagine that only few of the youngest children have an idea about two-digit numbers and would probably guess rather often. Is this a problem for the analyses?

We conducted this task (comparing single-digit [1-9] and two-digit verbal numbers [11-19]) with every child (including younger children and older children). To prevent the guesswork trend, all trials were presented in a fixed random order, according to four criteria: maximum three consecutive same-answer items, maximum two successive same-condition items, no following items of identical pair, and the first two items corresponded to far-small pairs (i.e., 2 – 5).

Preliminary analyses run on NW-Comparison tasks showed that performance was above chance level (*t*-tests indicates that mean performance is significantly above 12/24 for VN (*M* = 14.93, *SD* = 4.44): *t*(103) = 6.73, *p* < .001 and for BEL (*M* = 15.14, *SD* = 3.82): *t*(103) = 8.34, *p* < .001), Moreover, younger children of both countries perform above chance overall, VN: (*M* = 13.43, *SD* = 3.49): *t*(46) = 2.80, *p* = .007 and BEL: (*M* = 13.00, *SD* = 2.97): *t*(46) = 2.31, *p* = .026.

Because Reviewer # 2 requires the use of age as a continuous variable. In the new results section, we adjusted the analysis results by using age (in months) as a continuous variable instead of using the age as a between-subject variable group. Therefore all analyses are now run with age as a continuous variable.

\# Results

"In the first section, one-way ANOVA was conducted to compare children's cognitive performance (IQ and PL)." According to DFs, for PL the authors conducted an ANOVA (age x nationality).

We thank the Reviewer for the comment. Our updated analyses were run by ANOVA for I.Q. and ANCOVA for PL with nationality as between-subject variable and age as a covariate. Please see page 22.

Table 2: I would reiterate in a note that for addition/approximate addition N was smaller (because the youngest children didn't do the task).

Yes, we corrected that by showing *N* at each task. Please see Table 2, page 24.

Counting: Did the authors correct for multiple comparisons? Not sure if Figure 1 is needed.

Yes, we corrected for multiple comparisons for all eight numerical tasks. Please see the updated results section. We agree with the Reviewer about the figure (only showing the average score across nations) is unnecessary. Therefore, we removed three figures (1, 3, 4) and kept only figure 2 (page 27, line 646: → Fig 1. Cumulated percentage of children able to count up to at least that number in the counting list)

Enumeration: It remains unclear where the three-way interaction of set size, nationality, and age group comes from. I would suggest to not use the wording "performed non-significantly better" (in any of the sections).

You are right. This interaction was not revealing very interesting results. Furthermore, as we decided to run MANOVAs to decrease the number of analyses (as suggested by Reviewer #2), size is no more used as a variable of interest in the analyses.

Give-N: I don't think Figure 3 is necessary. Did the authors correct for multiple comparisons? The authors write that "The difference in number-knower level was significant from *two-knowers* to *three* *knowers* […]" although for three-knowers p=.051 (see also summary of results, discussion).

I also don't think Figure 4 is necessary (provides the same information as Table 4).

Yes. As mentioned earlier, figures 3 and 4 were deleted. After correcting for multiple comparisons for the Give-N task, it appears that the cardinality knowledge did not significantly differ across the two samples (*p* = .059). We only found a significant difference in *two-knowers* in post-hoc analyses (page 27-28, lines 650-655)

Number word comparison: line 576ff: Not sure if I fully understand the pattern here. I would like to ask the authors to check the means/sd presented in the text (I would be surprised if younger children performed better for the large set than older children).

While the authors show that both groups of children perform above chance overall, I am wondering if this is actually driven by the older children (and the younger children do perform on chance level). This is a bit hard to disentangle though because – as mentioned above - I am not fully convinced that the means are reported correctly.

We thank the Reviewer for this correct remark. We checked carefully for the mean, S.D., and *t*-test, and detected a mistake in the report of the mean between younger children for large size and older children for small size.

“the size effect was significant for younger children, small (*M* = 6.85; *SD* = 2.03) vs large (*M* = 6.36, *SD* = 1.84, instated of *M* = 9.00; *SD* = 2.44), *t*(206) = 6.93, *p* < .001 and for older children, small (*M* = 9.00; *SD* = 2.44 instated of *M* = 6.36; *SD* = 1.84) and large (*M* = 7.54; *SD* = 2.32), *t*(206) = 4.09, *p* < .001.”

As mentioned earlier, all analyses are now replaced by MANOVAs that do not include the size factor.

As regards the item analyses & summary of results: Did the authors correct for multiple comparisons here? A this point this is critical because when controlling, I expect the two now significant p-values to not be significant any longer. What is left for the number word comparison task is then a marginally significant interaction of set size and nationality which is not very conclusive.

Yes, we corrected for multiple comparisons (page 24-25)

To sum up, we found an age effect in all the tasks, showing that all of them were sensitive to the child's development. More importantly, we found significantly better counting performance in Vietnamese children (starting at teens numbers), but marginally better number cardinal knowledge in Belgian children (especially regarding the numbers two). For all the other measures, the performance of the children in both countries did not differ.

Questionnaire: Correction for multiple comparisons?

There were indeed too many analyses when considering each item separately. Instead, based on the suggestion of Reviewer #2, we analyzed only differences between formal and informal home numeracy and reported that in an exploratory results section (page 29-30, lines 686-709)

To sum, Vietnamese parents stimulated their child more on formal numeracy activities than Belgian parents. However, the frequency of informal numeracy activities did not differ between those of Vietnamese parents and Belgian parents. The Bayesians analysis also supported the significant difference in formal numeracy and the absence of difference in informal numeracy.

\# Discussion

Absence of an effect: "By contrast, the transparent, regular Vietnamese number-naming system did not facilitate the other numerical tasks." Inferential statistics cannot be used to provide evidence for a null effect. Specifically, in cases of (relevant) non-significant results, the authors might want to consider additionally using a Bayesian approach to strengthen their claim (can easily be done using, for example, JASP or JAMOVI). The authors also might want to consider discussion possible limitations due to sample size/power (while the sample is not small, ANOVAs include 2 between-group factors).

We thank the Reviewer for the suggestion. We used JASP for Bayesian analyses to test if the null hypothesis is true. Please read the updated results section (page 25-26)

The authors provide a comprehensive discussion of the observed results (depending on possible changes in the results section due to corrections for multiple comparisons the discussion might have to be updated).

Yes, we updated for discussion according to the updated results (please see p36, lines 809-811; p38, lines 869-871; p39, lines 878-889; p40-41, lines 919-923)

While I think the authors did a great job in trying to control for different cultural influencing factors (choosing Vietnam, assessing home numeracy) I think the interpretation of results might still have to be toned down a bit. While I think that the number word structure/morphology are plausible explanations, there might actually be other factors driving these differences that were not assessed (the factor in all presented analyses is called nationality and not language or number word structure or morphology). For example, differences in cardinality understanding might result from differences in morphology or from differences in home numeracy activities (where more informal activities might support cardinal knowledge acquisition). If the authors think differently, I would ask them to elaborate a bit more on how we know that it is indeed the number word structure/morphology that *causes* the observed differences.

We appreciate the positive assessment of our work. We agree with the Reviewer that there might be other factors driving these differences that were not assessed. With the updated way to analyses the home numeracy frequency, we found that Vietnamese parents stimulated their child more on formal numeracy activities than Belgian parents. However, the frequency of informal numeracy activities did not differ between those of Vietnamese parents and Belgian parents. We did not have the evidence to suggest that informal activities might support cardinal knowledge acquisition.

However, we see a contrast that, although Vietnamese children have the advantage of a more regular sequence of counting, and more frequent home numeracy (especially, formal home numeracy activities related to arithmetic or quantity/cardinality understanding), Vietnamese children are not better than Belgians children in understanding numbers (e.g., number word comparison task or give-N task). That is why we argue that morphology differences might be one possible explanatory factor for the difference in the Give-N task in particular. We also rely on evidence from previous cross-national studies to support a numerical morphology (i.e., singular-plural morphology, which exists in Western languages such as English, Russian, and French, but not in Asian languages such as Japanese, Mandarin, and Vietnamese) affects cardinality knowledge of small number words. Therefore, we added more detailed information about the morphology of the Vietnamese (page 10, lines 222-249) and remained our suggestion in the discussion.

\# data check

Important: Is ID a unique identifier for a child across all tasks (I was wondering about this because in the give-N task the N-level increases with I.D.)?

Advanced counting: only sum score (not item level data)

Collection comparison: only sum score per ratio (not item level data)

Addition: only sum score (not item level data)

Approximate addition: only sum scores per ratio (not item level data)

We thank the Reviewer for these remarks. We added the item-level data for all tasks. Please see the data file.

For Give-N task and all other excel sheets of data, there is only one I.D. for each child.

The Give-N data did not always increase with I.D., for example, VN47, Give-N = 6; VN48, Give-N = 1. We checked carefully for this data.

#small points:

Abstract: It is unclear what P.L. is

Yes, we corrected P.L. (Phonological loop) in abstract

p. 12, 295/303: highest?

Yes, the highest level of study. We explained this one above.

I would avoid unusual acronyms when they are not super necessary (N.W., C.R.)

Yes, We corrected that in all of the manuscript. We used the acronyms (N.W.) only in tables.

p. 29: "Correlations were then calculated between home numeracy activities for each sample separately and numerical performance for both groups."

According to the suggestion of Reviewer #2, moderation analyses were used to investigate the effect of nationality on the correlation between numerical abilities and home numeracy. Therefore, we corrected the way to report the results. Please see pages 31-32.

p.31 "Moreover, only the children who did not take extra mathematics courses outside of school were included in the study." -> move to sample description?

Yes. We corrected.

p. 38, l. 887ff: Yes, observed the exact same thing!

Yes. Although small, however, we observed 1 VN and 6 BEL children were successful for Give-5, but unsuccessful for Give-6. This issue is also reported in a recent article (Krajcsi et al., 2018) (lines 927).

Krajcsi, A., Fintor, E., & Hodossy, L. (2018). A refined description of preschoolers' initial symbolic number learning. https://doi.org/10.31219/osf.io/2kh9s
