# Supplementary material for: Transparent number-naming system gives only limited advantage for preschooler's numerical development: Comparisons of Vietnamese and French-speaking children
Source: PLoS One. 2020 Dec 7;15(12):e0243472. doi: 10.1371/journal.pone.0243472 (PMC7721146; doi:10.1371/journal.pone.0243472)
Supplement: Attachment — Submitted filename: Review2.docx [file pone.0243472.s013.docx]

As a general remark that I see that the authors put a lot of work into the revision of the manuscript and commented on all the points raised.

However, in some parts (particularly the newly added parts) it reads as if time ran out in the end. Clarity, reading flow (embedding of new paragraphs in the manuscript) and particularly also language aspects (grammar, spelling etc.) should be carefully checked.

Other than that, I have the following remaining comments:

\# I am happy with the methods section, the authors added missing details and transparently clarified open questions.

\# I think an advanced organizer at the end of the first paragraph of the introduction could help prepare the reader for what is coming in the introduction.

\# The section “The present study” is very long. The authors might consider shortening it (e.g., some of the sample information reappears in the methods section I believe) and/or structuring it more explicitly (e.g., subheadings; present the whole layout of the study at the beginning of the paragraph and then justify the design piece by piece).

\# Hypotheses

“We predicted that cross-cultural differences in home numeracy could also contribute to the difference between the two samples in the numerical tasks.” Either “predicted” or “could also contribute”. A prediction would be a confirmatory test, “could also contribute” points to an exploratory approach. Generally, I would ask the authors to check the formulations in the hypotheses section and be explicit about which are confirmatory and which are exploratory analyses.

\# level of difficulty

Manuscript: ”The analyses, including the level of difficulty of the items of the task (i.e., the ratio for collection comparison and approximate addition, size for enumeration, and number word comparison), did not show different effects of these variables between two samples and are thus not reported here.”

Response letter: “As you decided to run MANOVAs instead of multiple ANOVAs, the size effect was not included anymore in the analyses. This did not change much of the results as the previous analyses, including that factor, mostly failed to show any significant interaction between Nationality and Size.”

I wonder which version is the correct one. (If there were only minor effect maybe a better formulation would be “.. did not substantially change results..” or something similar

#Power

It is unclear to me how the power analyses were done (For which effect did the authors calculate power?) The details in the manuscript are not sufficient here. I tried to reproduce their estimates in Gpower but as far as I can see, it does not provide an option for MANCOVA, so I would be interested to see how the authors solved it.

Moreover, as the authors conduct post-hoc power analyses, it would be more intuitive in my view to report the effect size that could be detected with the current sample size.

I suggest asking the authors to provide the Gpower output and a precise clarification of their estimates.

“The power analysis indicated that a total sample of 65 children would be needed to detect medium effects (f2= 0.25) with 95% power using a MANCOVA with alpha at .05. Thus, it is unlikely that our negative findings can be attributed to a limited sample size.” No, this is exactly what is a possible explanation. If the effect of interest is small (smaller than .25) then the sample size might not be big enough to detect it.

#Bayes

A description of what Bayes factors mean and an explicit definition of what the authors (the source they are referring to) consider anecdotal, strong etc. would help the reader understand the reported results more readily.

\# ”good internal consistency”

I would refrain from describing internal consistencies <.80 as “good”, because they are indeed not particularly good. I would suggest the commonly used term “acceptable” and possibly discuss this as a limitation.

\# ”proved” is a word I would suggest avoiding ( I am not convinced that we can prove anything with a single study or even with multiple ones)

\# ”Besides, we provided additional evidence that the transparency of the number-naming system effect more robust the counting development of young children than home numeracy.” This is the final statement of the manuscript and I am wondering on which basis the authors draw this conclusion. Is the evidence for language indeed more robust (in the current study)? (Also, this sentence needs some additional words to be correct)

\# English proof reading

Especially the newly added parts need additional proof reading. To be quite honest, I don’t think the English proof reader (or maybe grammerly) improved the manuscript (rather, some more mistakes were added; e.g., p. 4, l. 123 the inclusion of “a”, p. 30, ;. 711 “decide”, p. 35, l. 804 “Let's us note(…)”)
