# Supplementary material for: Transparent number-naming system gives only limited advantage for preschooler's numerical development: Comparisons of Vietnamese and French-speaking children
Source: PLoS One. 2020 Dec 7;15(12):e0243472. doi: 10.1371/journal.pone.0243472 (PMC7721146; doi:10.1371/journal.pone.0243472)
Supplement: Attachment — Submitted filename: Response to Reviewers.docx [file pone.0243472.s014.docx]

**Transparent number-naming system gives only limited advantage for preschooler's numerical development: Comparisons of Vietnamese and French-speaking children.**

*Second revision*

*** Author's reply in blue**

Dear Editor and Reviewers,

Thank you for your consideration of our manuscript and many valuable propositions to improve our paper. We are happy to know that the paper considerably improved after the first revision.

We reply to the two points most important in the revised requirements and then ten minor requirements and questions from Reviewer #2

***First, Power analysis***

As regards the Reviewer's feedback that "*post-hoc power analyses are not very helpful and could be misleading because they do not reflect the true power of a study (simply the observed power, which is inversely related to the p-value"* and the propositions of the Editor "*remove these analyses and either replace them with power analyses based on previous estimates of relevant effect sizes in the literature (e.g., coming from meta-analyses or previous studies) or discuss the sample size limitation in the discussion*."

Our corrections are:

(1) Remove all previous post hoc analysis (please see the **tracked manuscript**: p.24, p.26, p.30)
(2) Add the discussion related effect size in addition (p.38, lines 855-869) and the effect-size limitation in cardinal knowledge (p.42, lines 953-960)

We compared our current study with three previous studies, which showed the consistent finding of group effect on counting and addition (supporting the higher performance of Chinese children) and on cardinal knowledge (supporting the higher performance of non-Asian children) (Please see Table 1 in page 2 of this response letter). Other abilities were not compared because of the inconsistent findings between different studies or have not been examined before (as discussed in the discussion section).

Except for counting ability, we found a consistent medium effect size in all three studies. For cardinal knowledge, although our sample (*N* = 208) was bigger than previous study (*N* = 136), however, we found the smaller effect size of group effect. We agree that it is a limitation of effect size. We recommended that future studies need to examine cardinal knowledge in younger children to favor the comparison.

For addition, Chinese kindergarteners engage in regular mathematics learning sessions (Ng & Rao, 2010), and even Chinese parents provided more frequent home numeracy than Vietnamese parents did (mean of 4 [very often] (Pan et al., 2006) vs. mean of 2 [sometimes] in the current study). Therefore, we suggested that the age difference (see Table 1, Vietnamese sample is younger than Chinese children, i.e., 57 months vs. 70 months) and sample selection (Vietnamese instead of Chinese sample) could explain the failure to find the group effect on addition in the current study, but not an insufficient sample size (*N* = 171 vs. *N* = 51).

| Table 1: Comparison of sample size and effect size between previous studies and the current study | | | | | | | | | | |
| --- | --- | --- | --- | --- | --- | --- | --- | --- | --- | --- |
| Abilities and studies | | | Groups | | *N* | | *n* | *M*_age_  [Range Age ] | Group effect | Effect size |
| *Counting* | | |  | |  | |  |  |  |  |
| Miller et al. (1995) | | | 2 | | 197 | | **99 Chinese**; 98 American | [3;2−5;9] | ***p* < 0.01** | *f =* 0.27*;* Medium |
| Lonnemann et al. (2019) | | | 2 | | 74 | | **37 Chinese**; 37 German | [4;1−6;2] | ***p* = 0.002** | *r =* 0.34; Medium |
| Current study | | | 2 | | 208 | | **104 Vietnamese;** 104 Belgian | 55 months  [3;5−5;5] | ***p* < 0.001** | *η2=* 0.146*;* Medium |
| *Addition* | | |  | |  | |  |  |  |  |
| Geary (1993) | | | 2 | | 51 | | **26 Chinese**; 25 American | 71 months | ***p* < 0.001** | *f* = 1.03*;* Large |
| Current study | | | 2 | | 171 | | 87 Vietnamese; 84 Belgian | 57 months  [4;0−5;5] | *p* = 0.580 |  |
| *Cardinality knowledge* | | |  | |  | |  |  |  |  |
| Sarnecka et al. (2007) | | | 3 | | 136 | | 29 Japanese; **39 Russian**; **68 American** | 3;2  [2;9−3;7] | ***p* < 0.01** | *f* = 0.82*;* Large |
| Current study | | | 2 | | 208 | | 104 Vietnamese; 104 Belgian | [3;5−5;5] | *p* = 0.059 | *η2=* 0.017 |
|  |  |  | |  | |  |  |  |  |  |

***Second, English proofreading***

For English proofreading (recommended by both reviewer #1 and 2), we corrected the style (more active voice), explicitness (removing any unnecessary words), length (rewrite more concise), reading flow, and carefully check the grammar and orthography. Then, we send the manuscript to a professional American (native English speaker) for the final proofreading. After received their correction, both authors confirmed the rereading of this final revised version. We hope this work can improve the manuscript's clarity to satisfy the requirements of Reviewers and Plos One.

We also corrected all other reviewers' suggestions in this second revision manuscript, shortened the introduction (present study), and explained how we computed the Power analysis for MANOVA (please see p.5 in this letter response to reviewer #2 and Output on Annex).

In addition, we also carefully checked the image (figure 1) and statistical reporting following Plos One's guideline. The ethics statement (p.12, lines 306-310) was added according to the requirement of Plos One (below).

***
1) Thank you for including your ethics statement on the online submission form:  "Ethics approval for this study was obtained from the Ethics Committee of Psychological Sciences Research Institute of the Catholic University of Louvain. Approval number: Projet2017-05. All children’s parents were given a written consent.". To help ensure that the wording of your manuscript is suitable for publication, would you please also add this statement at the beginning of the Methods section of your manuscript file.

**Reviewer 2:**

2. Based on my (limited) understanding of power analyses, I believe that post-hoc power analyses are not typically used or considered useful (see: http://daniellakens.blogspot.com/2014/12/observed-power-and-what-to-do-if-your.html#:~:text=Observed%20power%20(or%20post%2Dhoc,size%20estimate%20from%20your%20data.&text=Observed%20power%20is%20a%20useless,for%20post%2Dhoc%20power%20analyses.) Therefore, I recommend removing these from the manuscript and instead just including a brief note of this in the limitation section.

Yes. Thank Reviewer 2 for this insight and documents. I removed this analysis.

\# I am happy with the methods section, the authors added missing details and transparently clarified open questions.

Thank you for your appreciation!

\# I think an advanced organizer at the end of the first paragraph of the introduction could help prepare the reader for what is coming in the introduction.

Yes. We added this introduction (lines 68 to 71). The old section about the present study (lines 60-67) was removed and restructured in the present study section.

\# The section "The present study" is very long. The authors might consider shortening it (e.g., some of the sample information reappears in the methods section I believe) and/or structuring it more explicitly (e.g., subheadings; present the whole layout of the study at the beginning of the paragraph and then justify the design piece by piece).

Yes. We thank the Reviewer for these suggestions!

We shorted the present study section from 9 paragraphs to 6 paragraphs and restructured them (pages 8-10 in the manuscript without tracked). The structure of six paragraphs was:

(1) Overall aim
(2) Why running the study between Vietnam and Belgium
(3) Language characteristic of Vietnamese and French
(4) Precise objectives of testing
(5) Hypotheses
(6) Study design

The old paragraph related to numerical morphology (it is not very direct in this study) was deleted;

The sample selection (two big cities in Vietnam) was placed only in the methodology-sample section.

\# Hypotheses

"We predicted that cross-cultural differences in home numeracy could also contribute to the difference between the two samples in the numerical tasks." Either "predicted" or "could also contribute". A prediction would be a confirmatory test, "could also contribute" points to an exploratory approach. Generally, I would ask the authors to check the formulations in the hypotheses section and be explicit about which are confirmatory and which are exploratory analyses.

We thank the Reviewer for this insight. We checked for the formulations in the hypotheses section and determined that:

- The hypothesis related to Vietnamese children's higher performance in counting, enumeration, and simple addition is a confirmatory test.
- The hypothesis related to home numeracy is an exploratory test.

Therefore, we correct the words: predicted → assumed (p.12, line 293)

\# Level of difficulty

Manuscript:" The analyses, including the level of difficulty of the items of the task (i.e., the ratio for collection comparison and approximate addition, size for enumeration, and number word comparison), did not show different effects of these variables between two samples and are thus not reported here."

Response letter: "As you decided to run MANOVAs instead of multiple ANOVAs, the size effect was not included anymore in the analyses. This did not change much of the results as the previous analyses, including that factor, mostly failed to show any significant interaction between Nationality and Size."

I wonder which version is the correct one. (If there were only minor effect maybe a better formulation would be ".. did not substantially change results.." or something similar

Yes. We thank the Reviewer for bringing this point to our attention. We corrected this phrase in tracked manuscript*: We were unable to introduce the tasks' difficulty level (i.e., the ratio for collection comparison and approximate addition, size for enumeration, and number-word comparison) in the MANCOVA. However, previous analyses run separately on each task indicated that this did not substantially change the results.* (p.24, lines 569-573).

#Power

It is unclear to me how the power analyses were done (For which effect did the authors calculate power?) The details in the manuscript are not sufficient here. I tried to reproduce their estimates in Gpower but as far as I can see, it does not provide an option for MANCOVA, so I would be interested to see how the authors solved it.

Moreover, as the authors conduct post-hoc power analyses, it would be more intuitive in my view to report the effect size that could be detected with the current sample size.

I suggest asking the authors to provide the Gpower Output and a precise clarification of their estimates.

"The power analysis indicated that a total sample of 65 children would be needed to detect medium effects (f2= 0.25) with 95% power using a MANCOVA with alpha at .05. Thus, it is unlikely that our negative findings can be attributed to a limited sample size." No, this is exactly what is a possible explanation. If the effect of interest is small (smaller than .25) then the sample size might not be big enough to detect it.

Yes, we reply to the Reviewer two essential things related to Power analysis and your questions,

(1) **Compute Power Analysis for MANOVA.**

Yes. GPower does not provide options directly for MANOVA. However, we based on the reference: Chapter 6, Page 115, Example 10, GPower for MANCOVA from the book: Patrick Dattalo (2008). Determining Sample Size: Balancing Power, Precision, and Practicality. Oxford University Press. Please see the book shared in my google drive: <https://drive.google.com/drive/folders/19dotO9oTWOa-liNEgi9zO-ynqb9OoFOY?usp=sharing>

In this chapter, Dattlo (2008) explained that: MANCOVA is a MANOVA in which the dependent variables (DVs) are initially adjusted for differences in one or more covariates. Resources for estimating sample size for MANCOVA are challenging to identify. One approach to estimate the sample size for MANOVA is to use GPower and adjust the denominator df. If *k* is the number of groups in the design and *g* is the number of covariates, then groups = *k* + *g* (p.116).

(2) ***For which effect did the authors calculate power?***

I never did a power analysis before. The manuscript revision is one occasion to learn and practice this kind of analysis. I think that my estimates had some limitations.

First, I had a mistake reporting the estimates approach to power analysis. I did the priori power analyses; however, I wrote a post hoc analysis in the analysis plan. Besides, the sample-size reporting were inaccurate (*n* = 66 instated of *n* = 65; *n* = 90 instated of *n* = 91)

Second, I think in the first revision, I did not understand your requirement. The requirement #9 from reviewer 2: *While the sample size seems sufficient to detect at least a medium effect size within the analyses included (and is impressive!), it would be helpful to include a power analysis to indicate what exact effect sizes can be detected with each analysis using the current sample.*). So, Reviewer 2 asked for power analysis to compute the effect size, but I calculated the sample size.

Third, when I calculated the sample size required for three primary analyses of the paper (2 MANOVAs and 1 repeat-measure-related home numeracy), I introduced f^2^ = 0.12 or f^2^ = 0.15, or f = 0.15, f = 0.14 as the medium effect size (Please see in outputs annex), but after referred on Cohen (1998) and other resources, I know that it was not correct, the medium effect size required as f = 0.25. All previous power analyses did not make sense and were excluded according to the Reviewer and Editor's suggestion.

#Bayes

A description of what Bayes factors mean and an explicit definition of what the authors (the source they are referring to) consider anecdotal, strong etc. would help the reader understand the reported results more readily.

Yes. We added the definition of Bayes factor and the interpretation of their value (p.27, lines 632-636), according to Jeffreys, H. (1961). Theory of probability (3rd edition). Oxford: Oxford University Press, Clarendon Press.

#" good internal consistency"

I would refrain from describing internal consistencies <.80 as "good", because they are indeed not particularly good. I would suggest the commonly used term "acceptable" and possibly discuss this as a limitation.

Yes, we corrected as "acceptable" (p.31, line 719)

#" proved" is a word I would suggest avoiding ( I am not convinced that we can prove anything with a single study or even with multiple ones)

Yes. We thank the Reviewer for this suggestion! We deleted the word "*proved*" and rewrite the phrase (p.35, lines 776-777)

#" Besides, we provided additional evidence that the transparency of the number-naming system effect more robust the counting development of young children than home numeracy." This is the final statement of the manuscript and I am wondering on which basis the authors draw this conclusion. Is the evidence for language indeed more robust (in the current study)? (Also, this sentence needs some additional words to be correct)

No. Our evidence supported that both factors account for some part of the variance of young children's counting development. It is consistent with the previous study (Cankaya, LeFevre, and Dunbar, 2014). However, because we based solely on the Beta coefficient from regression analysis (Beta for nationality is higher than those for home numeracy), the evidence for *"transparency of the number-naming system effect more robust the counting development of young children than home numeracy"* is insufficient.

We conducted another regression to examine whether home numeracy was a significant predictor if we forced first the age and nationality into the model. The result showed that age, nationality, and home numeracy are significant predictors, and home numeracy still accounts for 5.5% of counting's variance. It was similar to the previous analysis (related to the section Language, home numeracy, and counting variation, p.34), which showed age, nationality, and home numeracy are significant predictors and nationalities accounted for 9.5% of counting's variance.

Our main study aims to assess the language's effect on numerical performance and control with home numeracy, but not compare these factors' strength. Therefore, we agree with the Reviewer's consideration and therefore removed this conclusion and replaced it by "*we extended the evidence that both the transparent number-naming system and home numeracy influence young children's counting development* " (p.43, lines 977-979 and also at abstract, p.2, lines 42-46). Thank the Reviewer for your careful assessment!

**Annexes**

*(Output related to sample size analysis; however, these analyses were eliminated according to the suggestion of Reviewer 2)*

- **Manova 1 (6 numerical task)**

N of groups = k (number of groups in the design) + g (number of covariates) = 2 groups (VN/ BEL) + 1 (covariate: age) = 3

n of responses (VD): 6

**F tests -** MANOVA: Global effects

**Options:**	Pillai V, O'Brien-Shieh Algorithm

**Analysis:**	A priori: Compute required sample size

**Input:**	Effect size f²(V)	=	0.1299435

α err prob	=	0.05

Power (1-β err prob)	=	0.90

Number of groups	=	3

Response variables	=	6

**Output:**	Noncentrality parameter λ	=	23.3898300

Critical F	=	1.8108946

Numerator df	=	12.0000000

Denominator df	=	166

Total sample size	=	90

Actual power	=	0.9024094

Pillai V	=	0.2300000

- **Manova 2 ( 2 addition tasks)**

N of groups = k (number of groups in the design) + g (number of covariates) = 2 groups (VN/ BEL) + 1 (covariate) = 3

n of responses (VD): 2

**Analysis:**	A priori: Compute required sample size

**Input:**	Effect size f²(V)	=	0.15

α err prob	=	0.05

Power (1-β err prob)	=	0.95

Number of groups	=	3

Response variables	=	2

**Output:**	Noncentrality parameter λ	=	19.8000000

Critical F	=	2.4435908

Numerator df	=	4.0000000

Denominator df	=	126

Total sample size	=	66

Actual power	=	0.9553431

Pillai V	=	0.2608696

- Repeat-measure MANOVA

**Output for nationality effect**

(Effect size f = 0.1499830 was calculated from η^2^ = 0.022)

**F tests -** ANOVA: Repeated measures, within factors

**Analysis:**	A priori: Compute required sample size

**Input:**	Effect size f	=	0.1499830

α err prob	=	0.05

Power (1-β err prob)	=	0.95

Number of groups	=	3

Number of measurements	=	2

Corr among rep measures	=	0.5

Nonsphericity correction ε	=	1

**Output:**	Noncentrality parameter λ	=	13.2270014

Critical F	=	3.9068490

Numerator df	=	1.0000000

Denominator df	=	144

Total sample size	=	147

Actual power	=	0.9507908

**Output for home numeracy effect**

**(**the Effect size f =0.1568125 was calculated from η^2^ = 0.024)

**F tests -** ANOVA: Repeated measures, within factors

**Analysis:**	A priori: Compute required sample size

**Input:**	Effect size f	=	0.1568125

α err prob	=	0.05

Power (1-β err prob)	=	0.95

Number of groups	=	3

Number of measurements	=	2

Corr among rep measures	=	0.5

Nonsphericity correction ε	=	1

**Output:**	Noncentrality parameter λ	=	13.2786865

Critical F	=	3.9128750

Numerator df	=	1.0000000

Denominator df	=	132

Total sample size	=	135

Actual power	=	0.9512798
